# Clinical Manifestations and Trend of Dengue Cases Admitted in a Tertiary Care Hospital, Udupi District, Karnataka

**DOI:** 10.4103/0970-0218.69253

**Published:** 2010-07

**Authors:** Ashwini Kumar, Chythra R Rao, Vinay Pandit, Seema Shetty, Chanaveerappa Bammigatti, Charmaine Minoli Samarasinghe

**Affiliations:** Department of Community Medicine, Kasturba Medical College, Manipal, Karnataka - 576 104, India

**Keywords:** Dengue, dengue hemorrhagic fever, dengue shock syndrome

## Abstract

**Background:**

India is one of the seven identified countries in the South-East Asia region regularly reporting dengue fever (DF)/dengue hemorrhagic fever (DHF) outbreaks and may soon transform into a major niche for dengue infection in the future with more and more new areas being struck by dengue epidemics

**Objective:**

To study the clinical manifestations, trend and outcome of all confirmed dengue cases admitted in a tertiary care hospital.

**Study Design:**

Record-based study conducted in a coastal district of Karnataka. Required data from all the laboratory confirmed cases from 2002 to 2008 were collected from Medical Records Department (MRD) and analyzed using SPSS 13.5 version.

**Results:**

Study included 466 patients. Majority were males, 301(64.6%) and in the and in the age group of 15-44 years, 267 (57.5%). Maximum number of cases were seen in 2007, 219 (47%) and in the month of September, 89 (19.1%). The most common presentation was fever 462 (99.1%), followed by myalgia 301 (64.6%), vomiting 222 (47.6%), headache 222 (47.6%) and abdominal pain 175 (37.6%). The most common hemorrhagic manifestation was petechiae 84 (67.2%). 391 (83.9%) cases presented with dengue fever, 41 (8.8%) dengue hemorrhagic fever, and 34 (7.3%) with dengue shock syndrome. Out of 66 (14.1%) patients who developed clinical complications, 22 (33.3%) had ARDS and 20 (30.3%) had pleural effusion. Deaths reported were 11(2.4%).

**Conclusion:**

Community awareness, early diagnosis and management and vector control measures need to be strengthened, during peri-monsoon period, in order to curb the increasing number of dengue cases.

## Introduction

Dengue made its debut as early as 1780, when Benjamin Rush described the condition as “break bone fever”. This hitherto unfamiliar infection has now grown to demand the attention of all public health care providers. A mosquito borne fast emerging viral infection manifesting in four serotypes capable of causing dengue fever (DF), dengue hemorrhagic fever (DHF), and dengue shock syndrome (DSS), poses an increasingly perilous situation due to lack of antiviral drugs or vaccine.(1) Worldwide nearly 2.5 billion people continue to live at risk of contracting the infection while 50 million cases and 24,000 deaths are estimated to occur in 100 endemic countries. This includes hospitalization of nearly half a million cases of DHF, of which 90% are children. Treated DHF/DSS is associated with a 1% mortality rate while mortality rate among untreated cases escalates to 20%.(2)

India is one of the seven identified countries in the South-East Asia region regularly reporting incidence of DF/DHF outbreaks and may soon transform into a major niche for dengue infection in the near future. The first confirmed report of dengue infection in India dates back to 1940s, and since then more and more new states have been reporting the disease which mostly strikes in epidemic proportions often inflicting heavy morbidity and mortality, in both urban and rural environments.(3) Several fatal forms of the disease i.e., DHF, DSS have been reported in India from time to time in Kolkata, Delhi, and Chennai.(4–7) Until mid-1990s, dengue was reported from only three of the four South Indian states, namely, Andhra Pradesh, Karnataka and Tamil Nadu. All the four serotypes of the virus have been in circulation and documented in Tamil Nadu.(8) Since then, Kerala too, has reported annual epidemics.

During all these epidemics, children <15 years of age were quite severely affected, but majority of infection occurred in active adults in the age group of 16–60 years.(9,10) Certain common signs and symptoms such as fever, headache, myalgia, arthralgia and bleeding manifestations have also been observed. However, few other studies have depicted differences in age and sex distribution and clinical presentation.(11–14) The present study was done to analyze the trend of the disease over the years along with the clinical features, complications, and outcome of cases admitted to a tertiary care teaching hospital, which provides speciality health care to neighboring four districts in Karnataka.

## Materials and Method

A record-based descriptive study was undertaken to determine the clinical profile and outcome of all patients admitted to a tertiary care teaching hospital at Manipal, Karnataka (Kasturba Hospital) with a diagnosis of dengue, DHF and DSS according to WHO protocol,(15) from January 2002 to December 2008. All standardized dengue enzyme linked immunosorbent assay (ELISA) IgM antibody positive (Pan Bio Kit - Australia) cases were included in the study. Data were collected using a pre-designed questionnaire and analyzed using Statistical Package for Social Sciences (SPSS version 13.5).

## Results

**Figure 1:**
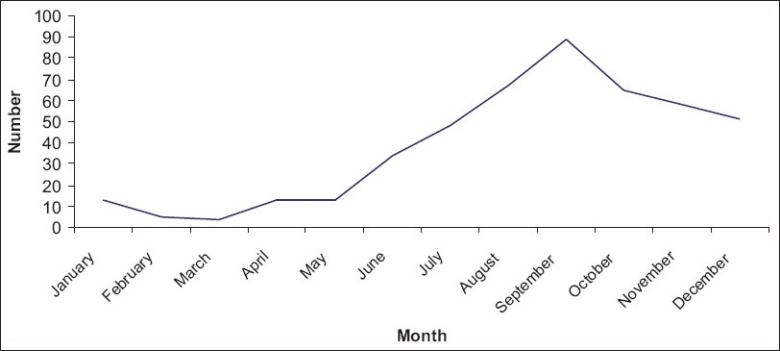
Month-wise distribution of dengue cases during 2002–2008 (*n*=466)

Of the total 466 cases, admitted to the hospital between 2002 and 2008, 391 (83.9%) had dengue fever, 41 (8.8%) had dengue hemorrhagic fever, and 34 (7.3%) had dengue shock syndrome. The year 2007 had the highest number of reported cases, 219 (47%). Most of dengue cases occurred during the month of September, 89 (19.1%) [Figure 1]. Table 1 depicts the districts from where the cases were reported predominantly, Davangere 192 (41.2%), Shimoga 107 (23%), and Udupi 35 (7.5%). Majority of the cases, 301 (64.6%) were males and 165 (35.4%) were females. Maximum number of cases was in the age group of 15–44 years, 267 (57.3%), while number of cases reported among the under-five children was 35 (7.5%). Average duration of stay in hospital was 6–10 days, 256 (54.9%). As seen in Table 2, fever was present in almost all cases 462 (99.1%) followed by myalgia 301 (64.6%), vomiting 222 (47.6%), headache 222 (47.6%), abdominal pain 175 (37.5%), breathlessness 83 (17.8%), diarrhea 65 (13.9%), skin rash 101 (21.7%), and altered sensorium 48 (10.3%). Hemorrhagic manifestations included petechiae 84 (67.2%), ecchymosis 29 (6.2%), gum bleeding 24 (5.2%), hematuria 23 (4.9%), malena 22 (4.7%), hematemesis 15 (3%), and epistaxis 12 (2.6%). In the study, 66 patients had complications of which, 22 (33.3%) patients had adult respiratory distress syndrome (ARDS), 20 (30.3%) had pleural effusion, 9 (13.6%) had multiple organ failure, 7 (10.6%) had encephalopathy, 4 (6.1%) had pneumonia, and 1(1.5%) had renal failure. The year-wise distribution of the deaths reported among 11 cases (2.4%) is depicted in Table 3. Of the 11 deaths, 7 (63.6%) were males, 4 (36.4%) were females and 7 (63.6%) were children.

**Table 1:** Socio-demographic characteristics of patients *n*=466

Characteristics	Number	Percentage
Age group (years)		
<5	35	7.5
5–14	72	15.5
15–44	267	57.3
45–60	72	15.5
>60	20	4.3
Sex		
Male	301	64.6
Female	165	35.4
Place of residence		
Davangere	192	41.2
Shimoga	107	23.0
Udupi	35	7.5
Other	132	28.3
Occupation		
Unskilled	144	30.9
Skilled	5	1.1
Professional	65	13.9
Preschool/school	138	29.6
Housewives	114	24.4

**Table 2:** Symptoms and complications of cases (*n*=466)

Symptoms	Number	(%)
Fever	462	99.1
Myalgia	301	64.6
Vomiting	222	47.6
Headache	222	47.6
Abdominal pain	175	37.6
Skin Rash	101	21.7
Petechiae	84	18
Breathlessness	83	17.8
Diarrhea	65	13.9
Altered sensorium	48	10.3
Ecchymosis	29	6.2
Gum bleeding	24	5.2
Hematuria	23	4.9
Malena	22	4.7
Hematemesis	15	3
Epistaxis	12	2.6
Complications		
Dengue with ARDS	22	33.3
Dengue with pleural effusion	20	30.3
Dengue and multi organ failure	9	13.6
Dengue encephalopathy	7	10.6
Dengue with pneumonia	4	6.1
Dengue with encephalopathy and ARDS	3	4.5
Dengue with renal failure	1	1.5

**Table 3:** Year-wise distribution of cases and outcome (*n*=466)

Year	Dengue fever	Dengue hemorrhagic fever	Dengue shock syndrome	Total no. of cases	Total no. of deaths
2002	6	0	1	7	0
2003	12	5	5	22	4
2004	10	0	2	12	1
2005	20	5	4	29	1
2006	39	11	5	55	1
2007	198	13	8	219	2
2008	106	7	9	122	2
Total	391	41	34	466	11

## Discussion

Dengue is an important emerging disease of the tropical and sub-tropical regions today. Since the first confirmed case of dengue in India, during the 1940s, intermittent reports of the infection and its sequelae have come from various parts of the country. These include reports from Ludhiana,(16) Delhi,(17–19) Lucknow,(11) Kolkata,(20,21) Chennai,(22) Mangalore,(23) Assam/Nagaland,(24) and Vellore.(25) The identification of dengue cases is possible by distinct clinical features but they can present with varied manifestations.(11–14) Research on dengue has grown exponentially, generating several specialized reviews.

Upon analyzing the year-wise distribution of dengue cases in the study population, steady increase in the number of dengue patients over the past few years was noted. Out of the total 466 cases, 219 (47%) were reported in the year 2007 whereas only 7 (1.5%) cases were reported in 2002. While this may be partially attributed to the rapid unplanned urbanization with unchecked construction activities and poor sanitation facilities contributing fertile breeding grounds for mosquitoes; it is also true that an increase in the alertness among medical fraternity following the initial epidemic and the availability of diagnostic tools in the hospital have contributed to the increased detection of cases.(26) Studies in Kerala parallel the situation of Karnataka as it has also shown a rise in cases from 1526 cases in 2004 to 2133 cases in 2006.(27)

To identify the seasonal variation of the disease, analysis of the data on monthly basis were done. A gradual increase in cases was noticed from June with a peak in September, during all the seven years of the study. Pre-monsoon increase in the number of cases was noted in the months of March and April which could be explained by the stagnation of water, after a few bouts of pre-monsoon rainfall which facilitate vector breeding. The correlation between occurrence of dengue and monsoon season is clearly evident in this study and is further supported by similar findings from Kerala,(27) Ludhiana,(28) Karachi.(29,30) These findings indicate that preventive measures against dengue infection should come into full swing during water stagnation periods after the initial bouts of rainfall and at the end of monsoon.

The male to female ratio in this study was 1.8:1 respectively. Congruent pattern was also seen in the retrospective analysis of the 2006 North Indian Dengue outbreak.(31) The study revealed that majority of the cases were in the age group of 15–44 years, 267 (57.3%). During the study, comparison between adults and children (<15 years) revealed that adults were infected disproportionately to that of children from 2002 to 2008. Although there was a dramatic increase in the total number of cases in 2007, this increase did not affect children. This pattern was also evident in the study conducted in Kerala. True endemicity will be reached when the adult infection declines and only the new entrants into the population, that is, the children, are affected more by the disease.(27) The clinical profile of dengue revealed that fever was the most common presenting symptom, 462 (99.1%). Similar studies in and around India have also substantiated fever as being the most common presenting symptom. Abdominal pain 175 (37.5%) and vomiting 222 (47.6%) were found to be present among 85.2% of the study population, which could be due to the liver injury caused by the dengue virus. It is imperative to keep in mind that other infections that cause fever and gastrointestinal symptoms such as typhoid, leptospirosis, enteroviral infections are common in India and may often lead to a delay in the diagnosis of dengue. Our study suggests that dengue in all its forms should be included in the differential diagnosis of patients with fever and gastrointestinal symptoms. This conclusion was also made from a study done in a tertiary care center in Pakistan.(29)

Hepatomegaly 248 (53.2%), altered sensorium 48 (10.3%), diarrhea 65 (13.9%), and skin rash 101 (21.7%) were not as frequent in our study as compared to other studies. An exclusive study on dengue shock syndrome conducted in Mumbai in 2003 reported hepatomegaly (97.4%), altered sensorium (58%), diarrhoea (50%), rash (42%), and cough (38%) in a significant number of cases. Headache was also seen less frequently compared to other studies.(32) Retro-orbital pain which is generally considered as a cardinal feature of dengue fever was not seen in our patients. Most of the patients presented with dengue fever (83.9%) while dengue hemorrhagic fever (8.8%) and dengue shock syndrome (7.3%) were a minority group. Similar findings have also been reported from rural Maharashtra.(33)

The most common bleeding manifestation in our study was petechiae 84 (18%), whereas during the 2006 outbreak of dengue in North India, malena (50%) and hematemesis (38%)(31) were found to be more common.

Of the 466 cases, 66 cases showed complications. ARDS was seen among 22 (33.3%) patients and pleural effusion among 20 (30.3%) cases. The overall outcome of patient care was good, with 455(97.6%) patients recovering completely. During the study period, 11 (2.4%) deaths were reported. Maximum number of deaths (4) were seen in 2003. Although there were a greater number of cases 219 (47%) in 2007, there were only two casualties probably owing to early diagnosis and prompt treatment.

The present study highlights the importance of dengue fever to clinicians in the areas of epidemiology, manifestations, complications and outcome of the disease. The study has the limitations inherent to a hospital record-based study, so meteorological and entomological data, information, education and communication (IEC) strategies and vector control measures initiated by the government are not correlated.
